# Highly porous and chemical resistive P(TFEMA–DVB) monolith with tunable morphology for rapid oil/water separation†

**DOI:** 10.1039/c8ra00501j

**Published:** 2018-02-22

**Authors:** Xiaozheng Wan, Umair Azhar, Yongkang Wang, Jian Chen, Anhou Xu, Shuxiang Zhang, Bing Geng

**Affiliations:** Shandong Provincial Key Laboratory of Fluorine Chemistry and Chemical Materials, School of Chemistry and Chemical Engineering, University of Jinan Jinan 250022 China chm_zhangsx@ujn.edu.cn chm_gengb@ujn.edu.cn

## Abstract

A facile preparation for a series of porous poly(2,2,2-trifluoroethylmethacrylate–divinylbenzene) P(TFEMA–DVB) foams is discussed in this paper. The foams have adjustable morphology utilizing a suitable commercial surfactant, Hypermer B246, as stabilizer, and were compared with traditional organic surfactants or macromolecular block-polymers. Combining the porous properties and advantages of fluorine atoms, this type of fluoropolymer exhibited superb chemical stability and hydrophobicity performances with high porosity. These porous fluoro-monoliths preserved their regular porous structure without any degradation after immersion into strong acidic or basic solution for three days, hence demonstrating an excellent potential to deal with environmental pollution caused by oil spillages in severe environments. The tunable morphology (open and closed pores) and pore sizes were achieved by investigating various parameters like surfactant concentration, amount of external crosslinker, and aqueous phase volume. Droplet sizes of HIPEs were characterized using an optical microscope under different experimental conditions. The influence of pore structure and surface properties of polyHIPE on water contact angle and oil adsorption capacity was also explored. The results indicated that the porous material has an excellent oleophilicity and hydrophobicity, with water contact angles (WCA) up to 146.4°. Additionally, the results presented a noticeable adsorption with a very fast rate towards organic oils from either a water surface or bottom with adsorption saturation achieved in about 120 s. The prepared polyHIPEs showed a good recycling ability; even after 10 adsorption–centrifugation experiments, the adsorption capacity was still more than 85%.

## Abstract 2

A facile preparation for a series of porous poly(2,2,2-trifluoroethylmethacrylate–divinylbenzene) P(TFEMA–DVB) foams is discussed in this paper.

## Graphical abstract

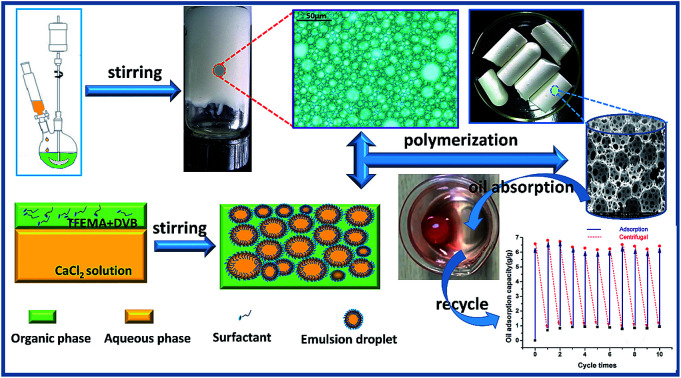

## 1. Introduction

Rapid oil/water separation is a challenging task during remediation processes with oil leakage accidents which cause serious problems to marine life and ecosystems.^1^ Oil adsorbents with high adsorption efficiency provide a good strategy for oil spill clean-ups. As of now, some traditional oil adsorbents such as kapok fiber, vermiculite, expanded graphite, graphite and zeolite have been applied for remedying an oil spill's damage to the environment.^2–5^ Due to some significant intrinsic shortcomings, for example, simultaneous adsorption of both water and oil, separation efficiency is lowered, so these inorganic materials for oil adsorption are not very useful in practical applications.^6,7^ Polymeric materials, especially porous polymers, have sparked remarkable interest as oil adsorbents from oil/water mixtures because of their large pore volumes, well-defined porosities, and interconnected structures. Because porous polymers combine the properties of both porous materials and polymers, they can also be used as scaffolds for tissue engineering,^8,9^ gas storage and separation,^10–12^ chromatographic column for separation sciences,^13,14^ supports for catalyst,^15–18^ and heavy metal ion collectors.^19–21^ Their potential applications have driven lots of research interests to develop reliable methods for preparation of porous polymers.

As for now, various methodologies have been developed to prepare porous polymeric materials,^22^ for example, molding or casting techniques, physical or chemical gas foaming methodologies, phase separation of block-polymers, and high internal phase emulsion methods. Using molding or casting techniques, a large number of porous polymers were prepared by monomer's polymerization in predesigned molds with subsequent removal of the templates. The limitation of this technology is that templates with predetermined porous structures prevent the adjustment of porous structures of polymers and also the mould's etching procedures are always challenging tasks for researchers.^23^ But, physical or chemical gas foaming techniques were efficient ways to get highly porous polymers. By using a high pressure gas directly or gas derived from chemical foaming agents, a typical close/open pore structure in a polymer was yielded in the extrusion foaming process of polymeric materials. However, practical use of this technique is hampered because of high temperature requirements, unavoidable use of organic solvents, and also equipment for a foaming process is relatively expensive as it must be strong enough to withstand high applied pressure and temperature parameters. Another major shortcoming of this technique is that pore connectivity and pore size adjustments of porous materials are not easy to achieve.^24^ Block-polymers are composed of two immiscible polymer chains connected by covalent bonds. With a phase separation technique, these incompatible segments can be driven to diverse areas by their thermodynamic incompatibility. After removing the sacrificial components of block-polymers, polymers with porous structures can be created.^25,26^ These methods also have some limitations in that the preparation of a block-polymer must be achieved using complex polymerization techniques, such as anionic living polymerization,^27^ atom transfer radical polymerization (ATRP),^28^ and reversible addition fragmentation transfer polymerization (RAFT).^29,30^ Furthermore, the pore sizes of these kinds of materials are always restricted to the nanometer scale.^27^

In 1984, the water-in-oil High Internal Phase Emulsion (w/o HIPE) template methodology was proposed by Lever Brother Company to prepare porous crosslinked polymeric materials.^31^ Since then, HIPE template methodology has been developed as one of the more effective and easy strategies to create macroporous polymer materials. Using HIPE templates, porous polymers with well-defined porosities and high specific surface areas were prepared and applied to various kinds of potential applications. HIPEs are highly viscous, paste-like emulsions having internal phase contents of 74% or more.^32^ In general, the continuous oil phase of HIPE (water in oil) undergoes polymerization, while the dispersion phase is mainly composed of microscale water droplets. By removing water droplets, a porous material after polymerization of the monomer–crosslinker mixture can be achieved. The sizes of droplets can be controlled easily by altering usage of a surfactant or by ratio of water/oil; thus, pore sizes of the porous materials are adjustable by a simple means through HIPE technology.^33^

Until now, many investigations have been carried out on polyHIPE materials based on polymerization of styrene (St) as a water-immiscible continuous phase and divinylbenzene (DVB) as crosslinker.^34^ In order to meet demands of different applications, induction of new functional monomers into HIPE systems were investigated, which significantly enhanced the properties of polyHIPEs. These kinds of monomers were mainly carbon–hydrogen compounds.^32,35^ But in some cases, porous polymeric materials have to be used under certain extreme conditions; for example, extreme weather conditions, high acid or basic service environments, *etc.* Porous polymers containing fluorine atoms have many positive impacts on their use in applications under harsh environments such as being highly acidic or basic. Fluorine atoms introduced into polymer chains, by virtue of their electronegativity, size, and bond strength with carbon, can be used to attribute polymers with extraordinary properties, such as low-surface-energy, anti-corrosion, thermal-stability, chemical resistance, *etc.*^36–38^

Yet, there is relatively less attention being paid on preparation of porous materials using fluorine-containing monomers.^32,34,35^ For fluoro-HIPE, it is very difficult to find a suitable conventional surfactant that can stabilize such kinds of emulsion systems. Even for common HIPE emulsions, *i.e.*, hydrocarbon-based, it should be noted that only a restricted number of conventional surfactants (*e.g.*, CTAB, Tween-80, Span-80, Span-60, *etc.*) are able to stabilize a particular monomer/aqueous system of emulsions.^19,34,39–42^

In our previous work, we synthesized a very efficient cationic fluorosurfactant diblock copolymer (PDMAEMA-*b*-PHFBA) to prepare a stable HIPE emulsion which gave rise to high performance fluoropolymer foams.^43^ As mentioned above, the synthesis of an amphipathic block-polymer, especially a fluoro-block polymer, was not an easy task. In this paper, a suitable commercial surfactant, Hypermer B246, was selected as stabilizer for preparation of a highly viscous and stabilized fluoro-w/o HIPE emulsion. By using this kind of commercial surfactant, a series of fluorinated porous materials were prepared with (2,2,2-trifluoroethyl methacrylate) (TFEMA) as a primary monomer, and divinylbenzene (DVB) as crosslinker. This prepared porous fluoropolymer demonstrated excellent hydrophobic and oleophilic properties along with specific porous and interconnected structures to rapidly adsorb organic oil from water. This type of porous fluoro-adsorbent provided good adsorption capacity and excellent reusability. It was interesting to find that the porous morphology of the foam could be simply and effectively tuned by the w/o ratio or by surfactant concentration. The significant properties of the porous fluoropolymer poly(TFEMA–DVB) were demonstrated by separating various types of oils from a water surface, indicating a promising application for treatment of environmental pollution such as oil spillages. The results showed that organic solvents were readily removed and reused after a simple adsorption–centrifugation step, which showcased excellent recyclability performance of these fluoropolymer monoliths.

## 2. Experimental

### 2.1 Materials

The monomer, 2,2,2-trifluoroethyl methacrylate (TFEMA, hydrophobic monomer), was purchased from Harbin Xeogia Fluorine-Silicon Chemical CO., Ltd. (Harbin, China), the cross-linker, divinylbenzene (DVB, technical grade, 80%), azobisisobutryonitrile (AIBN), and calcium chloride dihydrate (CaCl_2_·2H_2_O) were purchased from Sigma-Aldrich (USA). Span 80 (sorbitanmonooleate, HLB (hydrophilic–lipophilic balance) = 4.3), Span 85 (sorbitantrioleate, HLB = 2.1), Span 60 (sorbitan stearate, HLB = 4.7) were bought from Sigma-Aldrich (USA). Tween-80 (polyoxyethylene sorbitanmonooleate, HLB = 15.0), cationic surfactant CTAB (HLB = 15.8), anionic surfactant sodium dodecyl sulfate (SDS, HLB = 40), and Sudan III dyes were provided by Sinopharm Chemical Reagent Co., Ltd (Shanghai, China). Non-ionic surfactant Hypermer B246 (HLB = 4.9), a block copolymer of polyhydroxystearic acid and polyethylene glycol was supplied by Croda (USA). Toluene (>97%) was supplied by Tianjin Fuyu Fine Chemical Company (China).

The 2,2,2-trifluoroethyl methacrylate (TFEMA) and divinylbenzene (DVB) were filtered over basic alumina columns to remove the inhibitor and any other acidic impurities prior to use. Azobisisobutryonitrile (AIBN, ≥98%) was recrystallized from methanol and dried under reduced pressure for 12 h. Deionized water was used in all experiments. All other materials were used as received.

### 2.2 Preparation of polyHIPE materials

**Fig. 1:**
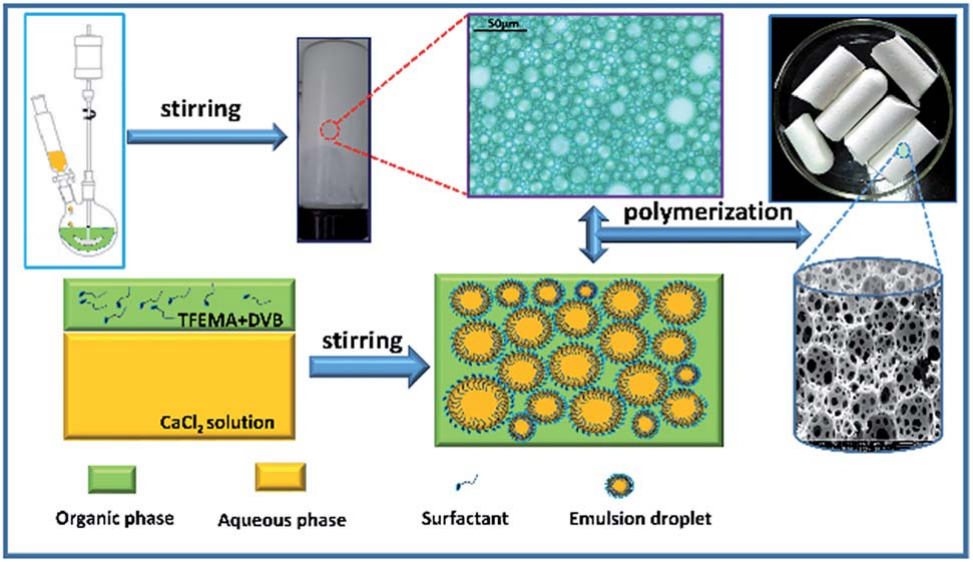
Schematic illustration of the fabrication of w/o HIPEs and highly porous fluoropolymer monolith.

A typical water-in-oil HIPE (sample C2) with 82 wt% aqueous internal phase was prepared as shown in Fig. 1. First, 2,2,2-trifluoroethyl methacrylate (TFEMA, 3.5561 g) and cross-linker divinylbenzene (DVB, 0.3951 g) were mixed in a glass vial. Then, the initiator 2,2-azobis-isobutyronitriermer (AIBN, 1 wt% to oil phase, 0.0395 g) and surfactants Hypermer-B246 (0.3951 g, 10 wt% to oil phase) were added to the monomer mixture and uniformly dispersed by ultrasonication (Hypation Branson Digital Sonifier) at room temperature for 3 min. Then, the mixture was placed in a 50 mL three-necked round-bottom flask equipped with an overhead controllable speed agitator. The aqueous phase of 0.2 mol L^−1^ calcium chloride solution (CaCl_2_·2H_2_O, 18 g) was added drop-wise to the oil phase with continuous mechanical stirring at 500 rpm. The speed of the droplets addition into the oil phase was set to 1 droplet per 3 s. Here, the CaCl_2_·2H_2_O as an electrolyte was added into the aqueous phase to suppress the effect of Ostwald ripening.^44^ The resulting HIPE was further agitated at 450 rpm for 30 min to ensure even distribution of emulsion droplets. Then, the prepared emulsion was transferred to a 10 mL centrifugal tube (polyethylene container) and put into an oven to polymerize at 70 °C for 24 h. Finally, the resulting monolith was dried until constant weight was achieved under vacuum at 60 °C. Other compositions of emulsions for polyHIPE preparation are listed in Table 1.

**Table 1:** Composition of high internal phase emulsion

Sample^a^	TFEMA/DVB^b^	Hypermer B246^c^ (wt%)	Internal phase fraction^d^ (wt%)	HIPE stability (days)
A1	2/8	10	82	>5
A2	4/6	10	82	>5
A3	5/5	10	82	>6
A4	7/3	10	82	>7
A5	8/2	10	82	>7
B1	9/1	5	90	>3
B2	9/1	10	90	>5
B3	9/1	20	90	>7
C1	9/1	10	86	>5
C2	9/1	10	82	>5
C3	9/1	10	78	>6
C4	9/1	10	74	>7

a For all the samples, AIBN was 1 wt% with respect to the total mass of the monomer and the cross-linker formed oil phase.

b Mass ratio of the monomer TFEMA and cross-linker DVB in oil phase.

c With respect to the total of oil phase.

d With respect to the total emulsion (the mass oil phase + aqueous phase).

### 2.3 Characterization

Emulsions were observed prior to polymerization using a light microscope Nikon (Eclipse LV100POL, Japan), equipped with a camera (Tucsen, model IS500). Microstructural studies of the samples were characterized with a field-emission scanning electron microscope (S-2500, Hitachi Seiki Ltd., Japan) equipped with an energy-dispersive spectrometer (EDS). Prior to SEM, approximately 0.25 cm^3^ pieces of monoliths were fixed on an aluminum stub with the help of carbon sticker and then sputtered with gold for 80 s (for better electron conductivity). The scanning electron microscopy images were further analyzed using Nano Measurer 1.2 software to obtain average pore and pore throat diameters. To compensate for the statistical error of the pore and pore throat sizes, at least 50 pores as well as 100 pore throats were measured, and a correction factor of 2/√3 was used to analyze SEM images. Porosity and density of the samples were calculated by the liquid displacement (*n*-hexane) test.^45^ The hydrophobicity and oleophilicity properties were measured through a Contact Angle Instrument (Data physics Co., Germany) at room temperature. 2 μL deionized water was dropped down each time, and then the water contact angle (WCA) values were recorded after 60 s of measurement. Thermogravimetric analysis (TGA) was characterized by a Pyris Diamond TG/DTA (PerkinElmer Co, USA) with a heating rate of 10 °C min^−1^ throughout a temperature range from 50 to 700 °C under nitrogen atmosphere. Openness is defined as the proportion of average diameter of interconnecting pores to average pore diameter. It is an important parameter for analyzing high interconnections between the pores when describing polyHIPE morphology. The typical equation used for calculation of openness is given as follows:^46^
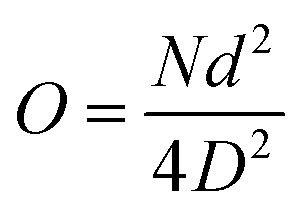
where *N* is the number of pore throats, *d* is average pore throat diameter, and *D* is average pore diameter. Pore and pore throat sizes were estimated by SEM image analysis using Nano Measurer 1.2 software. Specific surface areas of porous polyHIPEs were analyzed by utilizing an adsorption isotherm using the Brunaur–Emmett–Teller (BET) method. 200 mg of each sample was air dried for 12 h at 150 °C in a convection oven. Characterization was performed on a Micromeritics TriStar II 3020 (Quantachrome Instrument) at liquid nitrogen temperature. For the oil adsorption test, 0.15 g of poly(TFEMA–DVB) monolith foam (15 mm in height and 20 mm in diameter) in cylindrical shape was placed in a mixture of oil and water. As soon as the poly(TFEMA–DVB) monolith was removed from the oil/water mixture after oil adsorption saturation, it was weighed quickly to avoid evaporation of adsorbed organic liquids. Oil intake capacity *k* was calculated by the following equation:
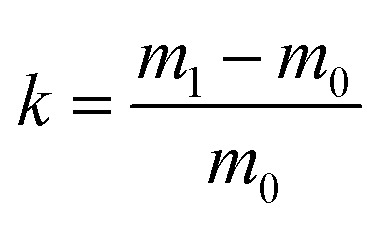
where *m*_0_ is mass of monolith before oil adsorption and *m*_1_ is mass of monolith after oil adsorption. Three replicates were performed, and the average value was then calculated for each sample.

## 3. Results and discussion

### 3.1 Selection of surfactant for fluoro-HIPE stabilization

The choice of a suitable surfactant to enhance thermodynamic stability and stabilize the emulsion system is of utmost importance. Type and concentration of surfactant have a great influence on the type of emulsion formed (o/w or w/o) and also on droplet size and structure. It is worth noticing that only a limited number of available surfactants are capable of stabilizing HIPE. At present, a few surfactants have been employed to stabilize emulsions using styrene as an organic continuous phase monomer but fluorinated monomers are difficult to emulsify, so it has been a challenge to synthesize high-performance fluoropolymer foams with controllable porous structures by HIPE due to lack of a suitable surfactant.

The stabilization of a particular system demands thoughtful selection of surfactant to achieve required hydrophobic–hydrophilic balance (HLB) for HIPE stability. A series of different HLB surfactants were carefully selected in order to stabilize w/o emulsions utilizing fluorinated acrylate as a continuous phase monomer. HIPEs were prepared from frequently used conventional surfactants, such as cationic surfactant CTAB, anionic surfactant SDS, nonionic surfactant, Tween 80, Hypermer B246, Span 80, Span 85, and Span 60 with the same process.

**Fig. 2:**
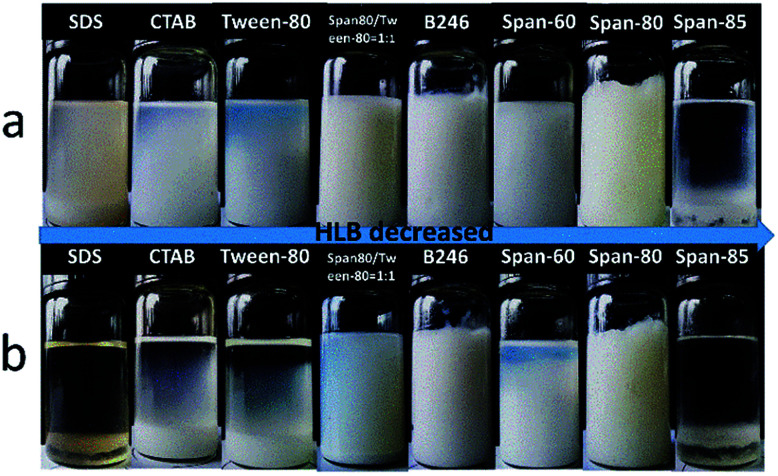
Photographs of w/o emulsions stabilized by different surfactants; (a) at time of preparation, and (b) after 1 hour at room temperature.

Fig. 2a shows photographs of emulsions prepared by different surfactants with 10 wt% concentration at room temperature after standing for 1 hour. It can be seen that cationic surfactant CTAB, anionic surfactant SDS, nonionic surfactant Tween 80, Span 85, and Span 60 were unable to stabilize emulsions. Clear bilayer phase separation occurred after standing the emulsion for 1 hour at room temperature (Fig. 2b). Span 80 gave some stability to the emulsion but failed to form porous materials after polymerization. Only Hypermer B246 proved to be best for such emulsions and also gave rise to porous material after polymerization, so that was selected as a suitable surfactant for further investigation.

### 3.2 Effect of amount of external crosslinker

**Fig. 3:**
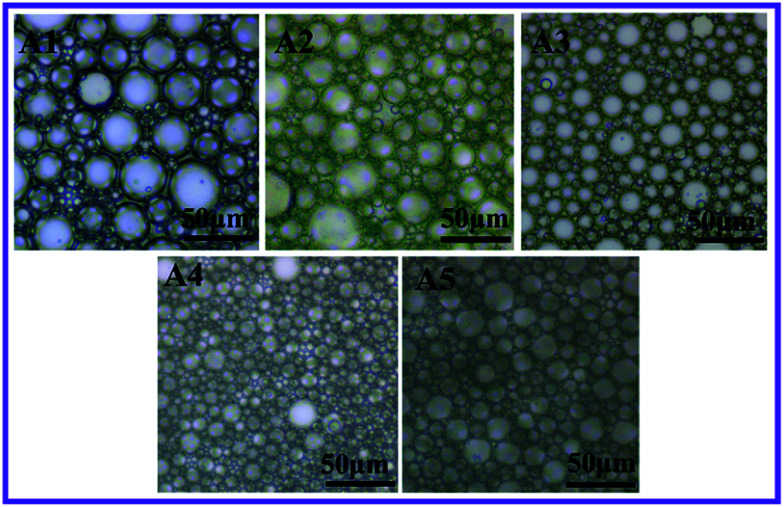
Optical microscopy images of w/o HIPE with different weight ratios of DVB to TFEMA (A1, 8 : 2), (A2, 6 : 4), (A3, 5 : 5), (A4, 3 : 7), and (A5, 2 : 8).

Usually, porous morphology of polyHIPEs can be adjusted by varying contents of the hydrophobic cross-linker.^47,48^ Here, for fluoroHIPE emulsions, DVB contents of an oil phase were varied at 80 wt%, 60 wt%, 50 wt%, 30 wt%, and 20 wt% relative to oil phase (Table 1, sample A1–A5), with the same surfactant concentration and aqueous phase fraction. Optical microscopy images (Fig. 3, A1–A5) show obvious differences in droplet sizes, deformation of droplets, and varying thickness of the oil layer between adjacent droplets among these samples. By gradually reducing contents of DVB from sample A1 to A3, a distinct reduction of droplet sizes of HIPE was observed. When the DVB contents were further reduced, the droplet sizes of emulsions started to increase from samples A3 to A5.

**Fig. 4:**
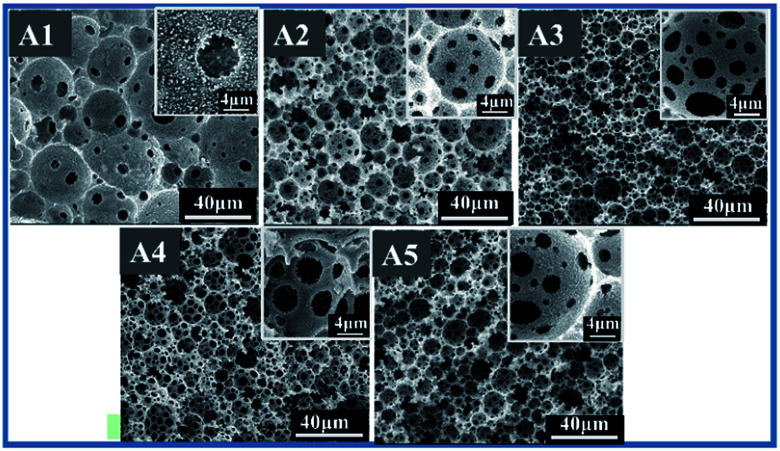
SEM images of polyHIPE with different weight ratio of DVB to TFEMA (A1, 8 : 2), (A2, 6 : 4), (A3, 5 : 5), (A4, 3 : 7), (A5, 2 : 8).

It is to be noted that, when contents of DVB were relatively less in the main HIPE formulation then the number of water droplets became high, thus the thickness of the monomer layer between droplets became less from sample A1 to A5 as shown in Fig. 3. Consequently, the pore throats were more likely to form with shrinkage of the monomer layers between adjacent droplets (Fig. 4). Although the phenomenon of pore throats formation is still under debate, it is believed that at variable concentrations of organic and/or internal phases, the layer of the oil between the water droplets thins and starts to shrink back. At points where the internal phase droplets touch each other they impart small openings known as pore throats.^43^Table 2 shows various parameters of porous fluoropolymers, typical average void diameter, throat diameter, number of pore throats per pore, density, porosity, openness and BET surface area of macroporous polymers; Fig. S1† demonstrates BET nitrogen adsorption–desorption isotherms for sample A5. Samples A1–A5 (Fig. 4) show distinct differences in pore sizes and interconnectivities. Pore and pore throat sizes of the normal distribution curves of samples A1–A5, and the distribution histogram of samples A5 are shown in Fig. S2† by Software processed SEM images. Average pore sizes of sample A3 were smaller than other samples due to the small emulsion droplets sizes. However, sample A5 had smaller average pore throat size resulting from comparatively smaller distances between adjacent emulsion droplets.

**Table 2:** Foam average void diameter (*D*_v_), throat diameter (*D*_t_), number of pore throats per bigger pore (*N*_t_) and density (*D*_m_), porosity (*P*), and openness (*O*_p_) of macroporous polymers

	*D*_v_ [μm]	*D*_t_ [μm]	*N*_t_	*D*_m_ [g cm^−3^]	*P* [%]	*O*_p_ [%]	BET surface area (m^2^ g^−1^)
A1	24.25	6.14	3.2	0.1531 ± 0.05	77.03 ± 2.1	3.53	9.94
A2	15.74	3.77	9.2	0.1392 ± 0.02	78.34 ± 1.8	13.48	24.08
A3	9.22	3.68	14.5	0.1253 ± 0.09	82.21 ± 1.2	59.14	27.17
A4	9.79	2.85	12.4	0.1273 ± 0.08	80.91 ± 1.3	26.06	24.35
A5	11.17	2.37	10.1	0.1356 ± 0.06	79.23 ± 2.1	11.93	15.39

From SEM results it can be concluded that too high contents of DVB are more likely to generate large and closed pores. Average void size of A3 was approximately 9.22 μm and average interconnecting pore size came out to be approximately 3.68 μm as compared to 11.17 μm and 2.37 μm for A5. This morphological variation may be attributed to the different reactivity and thermodynamic properties of the monomers involved in this fluoro-HIPE system.

It is also clear from Fig. 4 that the sizes of the pores decreased first and then increased with the change in DVB to monomer ratio. The pore sizes of polyHIPEs were in good agreement with the droplet sizes of the original emulsions, indicating that the coalescence of adjacent droplets was greatly suppressed during the polymerization process. Numbers of open throats were significantly fewer in the SEM image of sample A1, and these increased by decreasing DVB to TFEMA ratios until sample A5. One possible reason is that by decreasing the DVB fraction in the main HIPE formulation, the oil layer between two adjacent water droplets became too thick to be torn up during HIPE formation and polymerization as illustrated in optical images in Fig. 3 A1–A5 and SEM images in Fig. 4 A1–A5, respectively.

### 3.3 Effect of surfactant concentration

Surfactant concentration has proved to be one of the most important parameters in tuning pore structure and interconnectivity of polyHIPE.^44^ Macroporous polymers B1, B2, and B3 (in Table 1) were prepared with varying surfactant concentration at 5 wt%, 10 wt%, and 20 wt%. Water to oil ratio was constant at 90/10 and DVB concentration was constant at 10 wt% with respect to the continuous phase that was used.

**Fig. 5:**
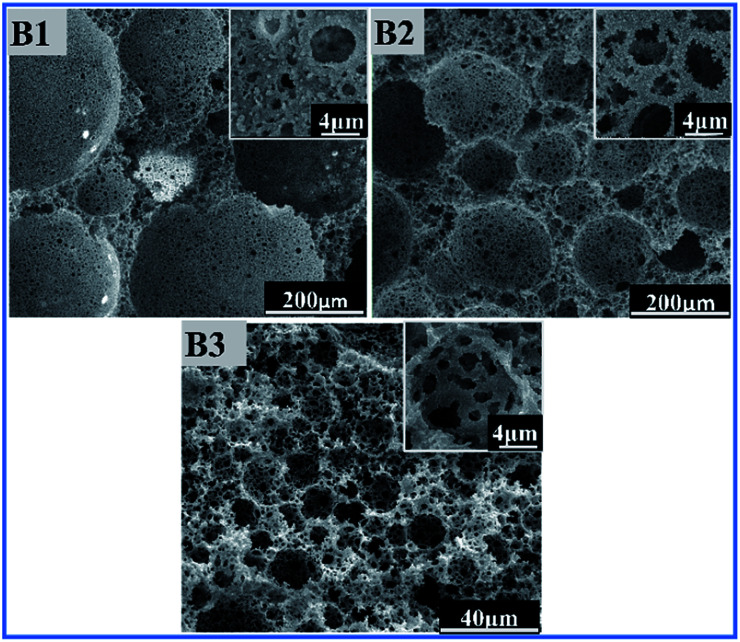
SEM images of polyHIPE with different content of surfactant to oil phase (B1) 5 wt%, (B2) 10 wt%, (B3) 20 wt%.

A clear-cut difference in pore size and morphology can be seen in SEM images (Fig. 5). Foam related test results are exhibited in Table S1.† When the surfactant concentration was low, only larger pore sizes of about 266.4 μm were formed. However, samples B2 and B3 displayed mostly irregular interconnected pores due to significant continuous phase volume contraction and film rupture in the polymerization process or post-polymerization treatment. This also resulted in formation of significant interconnections in the inner walls of the pores. As it can be clearly observed in Fig. 5 B1–B3 numbers of pore throats are higher in samples with relatively higher surfactant concentrations.

The SEM results were consistent with corresponding optical microscopy images shown in Fig. S3† and results are listed in Table S1.† A distinct decrease of droplet sizes can be observed when the surfactant content was increased from 5 wt% to 20 wt%. The possible reason for a decrease in pores diameters is due to the increase of HIPE droplet sizes because of coalescence between many small droplets. This means that by heightening concentration of surfactant to a certain level, coalescence decreases between droplets of emulsion which ultimately hinders the size that they can grow to and results in smaller size pores.

### 3.4 Effect of water/oil ratio on porous structure

Aqueous phase volume also has a strong impact on controlling morphology of polymers. Three HIPE samples C1–C3 (in Table 1) were prepared with various internal phase fractions of 74 wt%, 82 wt%, and 86 wt%. Both DVB concentration and surfactant concentration were kept the same at 10 wt%. The samples were observed by optical microscopy to characterize shapes of the dispersed droplets in HIPEs.

**Fig. 6:**
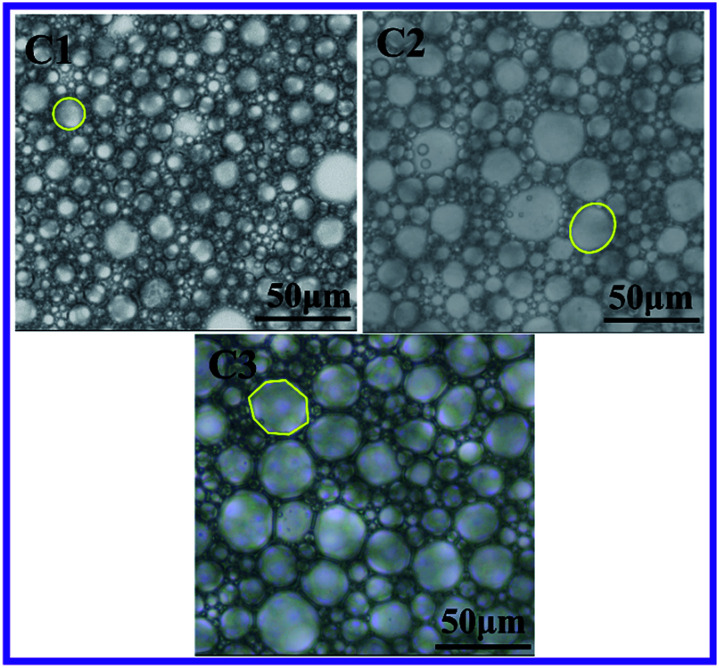
A clear illustration of transformation of droplet shapes from spherical to polyhedral *via* optical microscopy images of w/o emulsion with different aqueous phase weight percentage (C1) 74 wt%, (C2) 82 wt%, and (C3) 86 wt%.

As shown in Fig. 6, it is obvious that the shapes of droplets changed from spheres to polyhedral when the internal phase fraction increased from 74 wt% to 86 wt%. This was attributed to the distinct deformation of the droplets which was caused by increasing compressive forces between the growing quantities of water droplets on each other in a fixed space.

From the optical images, the average droplet sizes increased accordingly, and distribution of droplet sizes displayed a growingly uneven trend with addition of the aqueous phase fraction. This trend can be attributed to the phenomena of Ostwald ripening. As all three samples were prepared using the same amount of surfactant, hence the increment of internal phase fraction caused the surplus amount of droplets which were available for the surfactant molecules in the HIPE system. Hence, the limited surfactant molecules couldn't provide enough stabilization effect for the numerous small water droplets. These results are consistent with previous literature reports in which it was proved that larger droplets were more energetically favored than smaller droplets through a thermodynamically-driven spontaneous process if the aqueous phase fraction was maintained at a relatively high level.^49^

**Fig. 7:**
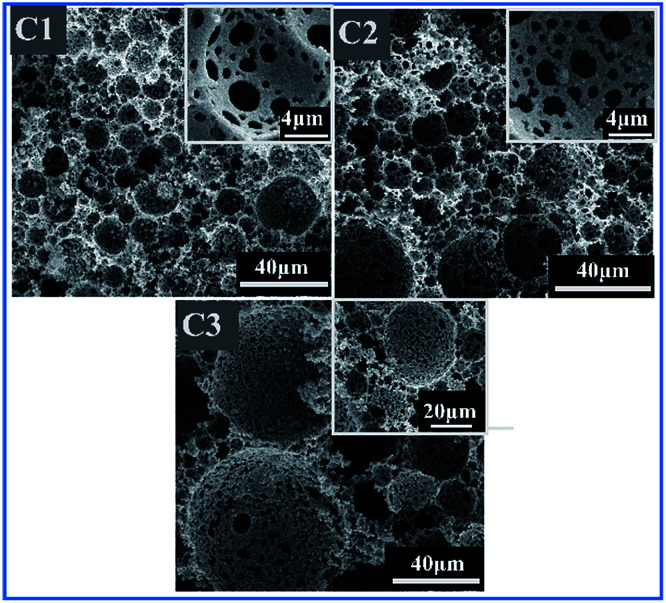
SEM images of polyHIPE with different aqueous phase fractions; (C1) 74 wt%, (C2) 82 wt%, and (C3) 8 6 wt%.

After polymerization, pore size and its distribution also exhibited similar results compared with initial results of the HIPE droplets. The average diameter of pores increased from 12.16 μm to 52.71 μm with the increase of water/oil weight ratio (Table 3, Fig. 7). The BET test result shows that specific surface area decreased from 18.12 m^2^ g^−1^ to 12.22 m^2^ g^−1^ by increasing aqueous phase contents.

**Table 3:** Foam average void diameter (*D*_v_), throat diameter (*D*_t_), number of pore throats per bigger pore (*N*_t_) and density (*D*_m_) of macroporous polymers

Sample	*D*_v_ [μm]	*D*_t_ [μm]	*N*_t_	*D*_m_ [g cm^−3^]	BET surface area (m^2^ g^−1^)
C1	12.16	2.81	16.4	0.1858 ± 0.05	18.12
C2	22.27	2.80	20.1	0.1762 ± 0.05	14.47
C3	52.71	2.72	—	0.1633 ± 0.05	12.22

From the SEM images (Fig. 7), more open throats were observed in corresponding porous polymers when the internal phase fraction was increased. This can be attributed to the thinning of monomer layers at a high internal phase fraction which leads to the formation of interconnected structure.^49^

### 3.5 Hydrophobicity and oil adsorption ability of P(TFEMA–DVB) foam

**Fig. 8:**
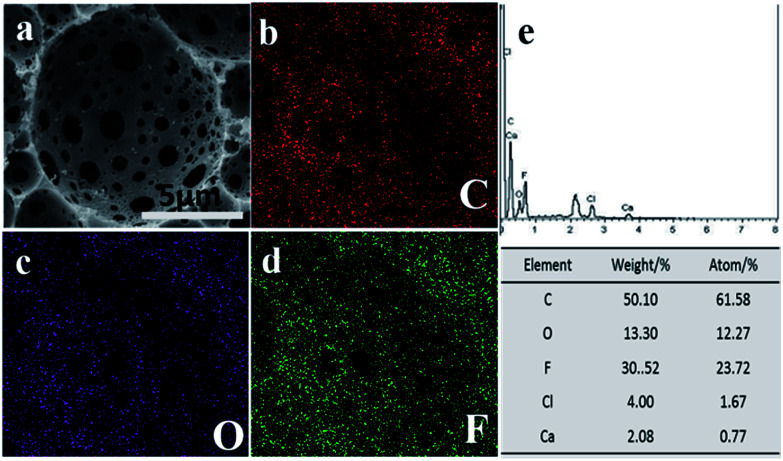
EDS analysis of polyHIPE A4; (a) SEM image and elemental mapping corresponds to (b) carbon, (c) oxygen, (d) fluorine; and (e) shows elemental contents in graphical and tabular form.

Porous polymeric materials with excellent hydrophobicity and oleophilicity exhibit high oil/water separation efficiency. The first requirement for a good oil sorbent material is that its surface should be composed of low-surface-energy materials. Fluoropolymers, such as poly(tetrafluoroethylene) (PTFE) and poly(vinylidene fluoride) (PVDF), provide efficient hydrophobic and oleophilic surfaces because of the low surface energy of the –CF_2_– group.^50,51^ For prepared P(TFEMA–DVB) foams, the EDS results of sample A4 indicated that fluorine atoms were mainly dispersed on the pore's surface (Fig. 8). The –CF_3_– groups of TFEMA monomer migrated to the porous surface of the material by a thermodynamic drive.^52^

**Fig. 9:**
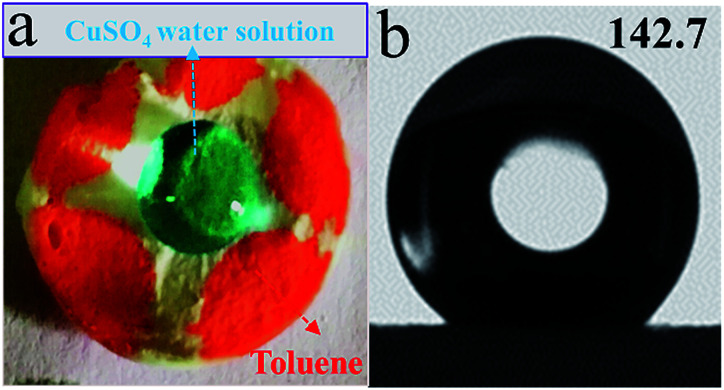
Sample A4 (a) hydrophobicity (CuSO_4_ water solution) and oleophilicity (toluene colored with Sudan III) demonstration; (b) Water Contact Angle (WCA) test.

Hydrophobicity and oleophilicity properties of sample A4 were analyzed by a Water Contact Angle (WCA) instrument. As shown in Fig. 9a, when a drop of water was dripped on the surface of the porous material, it did not penetrate into the pores, but rather turned into a spherical shape on top of the surface. Conversely, as soon as a globule of toluene (dyed with Sudan III for clear observation) touched the surface of the porous material, it immediately adsorbed into the material. The WCA of sample A4 was 142.7° as shown in Fig. 9b, which demonstrated its excellent hydrophobic nature.

**Fig. 10:**
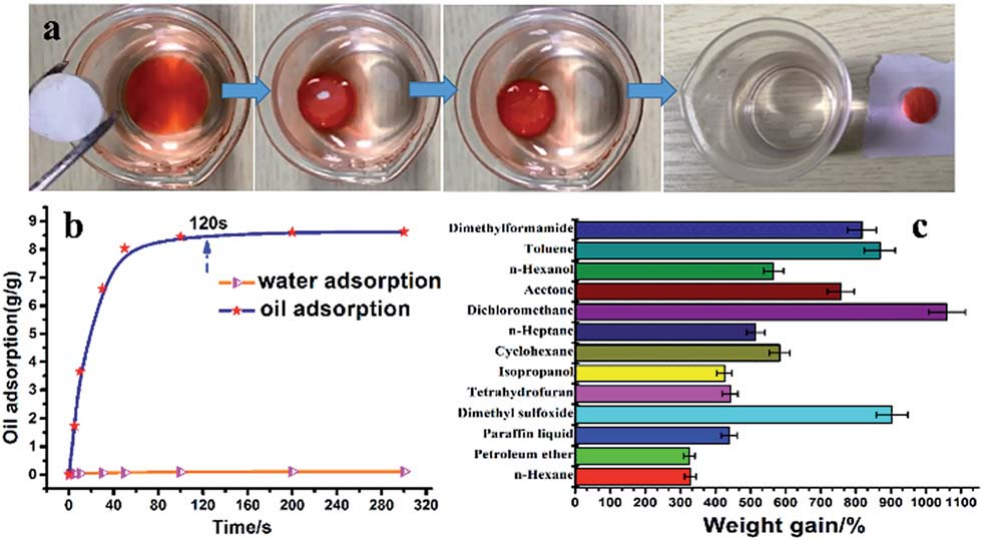
Sample A4; (a) process of oil adsorption, (b) oil adsorption rate curve, (c) adsorption capacity of the as-prepared polyHIPEs for various organic solvents.

P(TFEMA–DVB) foams proved to be excellent adsorption materials with special wettability according to the experimental data mentioned above. The oil adsorption test was performed to testify the adsorption performance of the P(TFEMA–DVB) monolith. Due to the excellent hydrophobicity, oleophilicity, and open cell structure, porous fluoro-polyHIPEs foams exhibited faster adsorption rates compared with reported adsorbents.^53,54^ The oil adsorption capability of P(TFEMA–DVB) adsorbents is demonstrated in Fig. 10 and ESI Video S1† (toluene dyed with Sudan III was used as reference oil).

The rate of oil adsorption was very fast; it took only 120 seconds to reach saturation equilibrium (Fig. 10b). The intrinsic hydrophobic and oleophilic surfaces of the prepared foams contributed high efficiencies for oil/water separation. Whereas, highly interconnected pores existed inside the monolith provided an ideal channel for oil transport inside the porous structure, which accelerated the adsorption rate.

Petroleum ether, *n*-hexane, paraffin liquid, isopropanol, cyclohexane, *n*-heptane, dichloromethane, acetone, *n*-hexanol, toluene, and so on were used as target oils to test the adsorption capacities of the foam (sample A4). As shown in Fig. 10c, capacities of foam A4 towards thirteen organic liquids were 3.5–10.5 times of its original mass. Another important feature of P(TFEMA–DVB) was that, even for organic solvents having higher densities than water, such as dichloromethane, these porous fluoro-polyHIPEs selectively and rapidly adsorb such oils from the bottom of the water (ESI Video S2†). It is clear from the video that when submerged in oil water mixture (where oil was on the bottom), that fluoropolymer foam selectively and rapidly adsorbed oil rather than water.

**Fig. 11:**
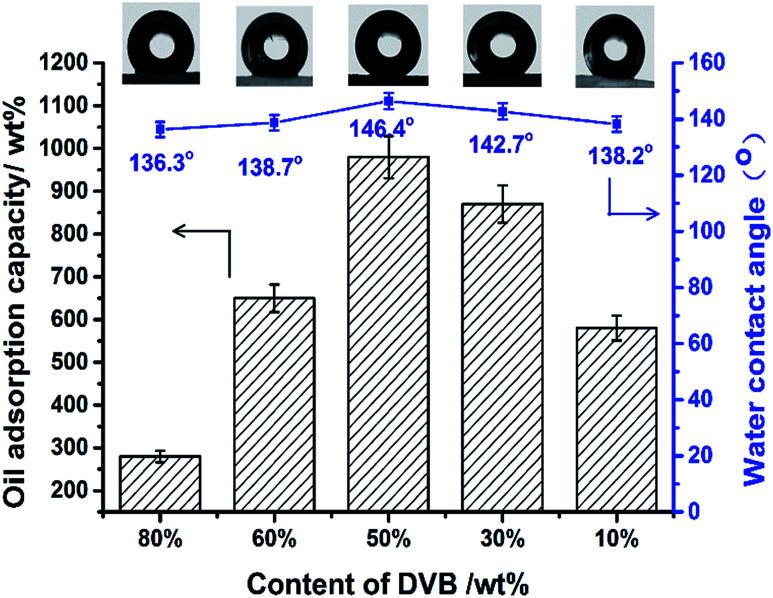
Oil adsorption capacity and water contact angle of fluoro-polyHIPEs with different contents of DVB.

To further verify the effects of surface properties on oil adsorption capacity, monoliths with different DVB amounts were prepared. From Fig. 11, oil adsorption capacity was affected clearly by the hydrophobicity of oil adsorbents, which was proved by water contact angle measurements. Increasing WCA means that the provision of more space for oil to penetrate into the pores, as water will be repelled at the same time due to the inherent nature of fluorinated porous monoliths. At 50 wt% DVB contents the highest value of WCA was achieved. Its oil adsorption capacity for toluene reached to 9.8 times that of the initial weight of monolith.

### 3.6 Chemical resistance and thermal stability of fluoro-monolith

Selected samples (A3, C2) were dipped into acidic and basic aqueous solutions to test chemical resistance of the prepared fluoropolymers. Clips were used to ensure good sinking of samples into solution.

**Fig. 12:**
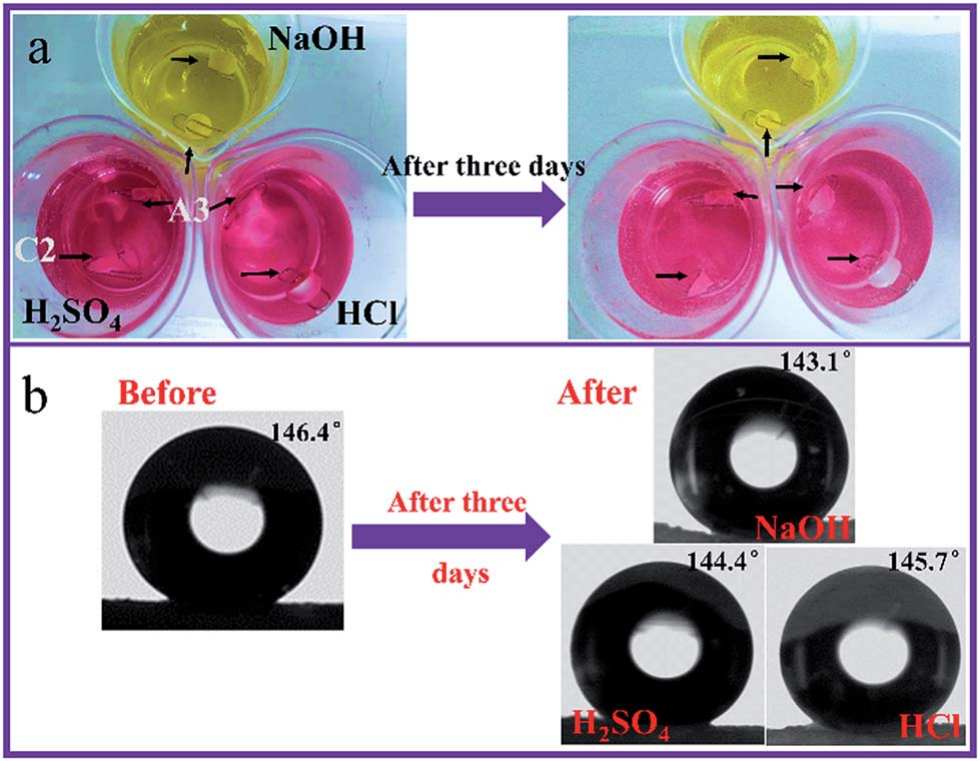
Acid and alkali resistance test (sample C2; DVB/TFEMA = 1 : 9, sample A3; DVB/TFEMA = 5 : 5): (a) immersion of samples in alkali (1 mol L^−1^ NaOH solution dyed with methyl orange) and acids (1 mol L^−1^ H_2_SO_4_, and 1 mol L^−1^ HCl solution dyed with methyl orange). (b) WCA for A3 before and after immersion of samples in chemical environment for 3 days.

**Fig. 13:**
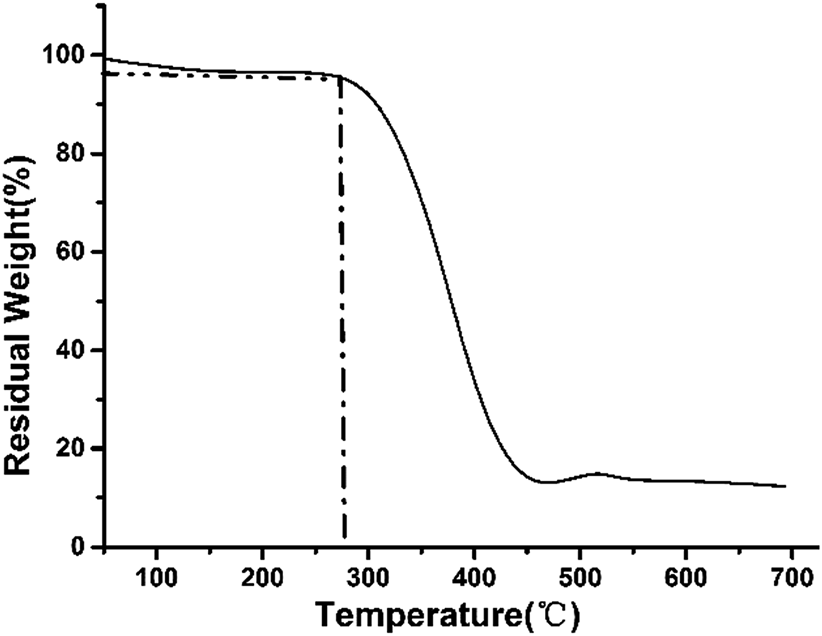
TGA curves of sample C2.

From Fig. 12a we can see that there was no obvious swelling or any significant damage to the surface of samples after immersion in acidic or basic solution for three days. Also, when sample A3 was immersed into 1 mol L^−1^ NaOH solution, 1 mol L^−1^ H_2_SO_4_, and 1 mol L^−1^ HCl solution for three days, water contact angles slightly dropped from 146.4° to 143.1°, 144.4°, and 145.1°, respectively. This suggested good solvent resistance and withstanding ability of the materials containing fluorine, even after being exposed to severe environmental conditions (Fig. 13).

Thermal stability of synthesized porous fluoropolymers was characterized by thermal gravimetric analysis (TGA). Here, temperature for 4 wt% loss was used as degradation temperature to estimate the thermal stability performance. Results of (TFEMA–DVB) porous materials showed remarkable stability performance at about 280 °C. This shows that the porous polymers prepared in this report have superb ability to be used under high temperature conditions.

### 3.7 Application and reusability of the P(TFEMA–DVB) porous material

**Fig. 14:**
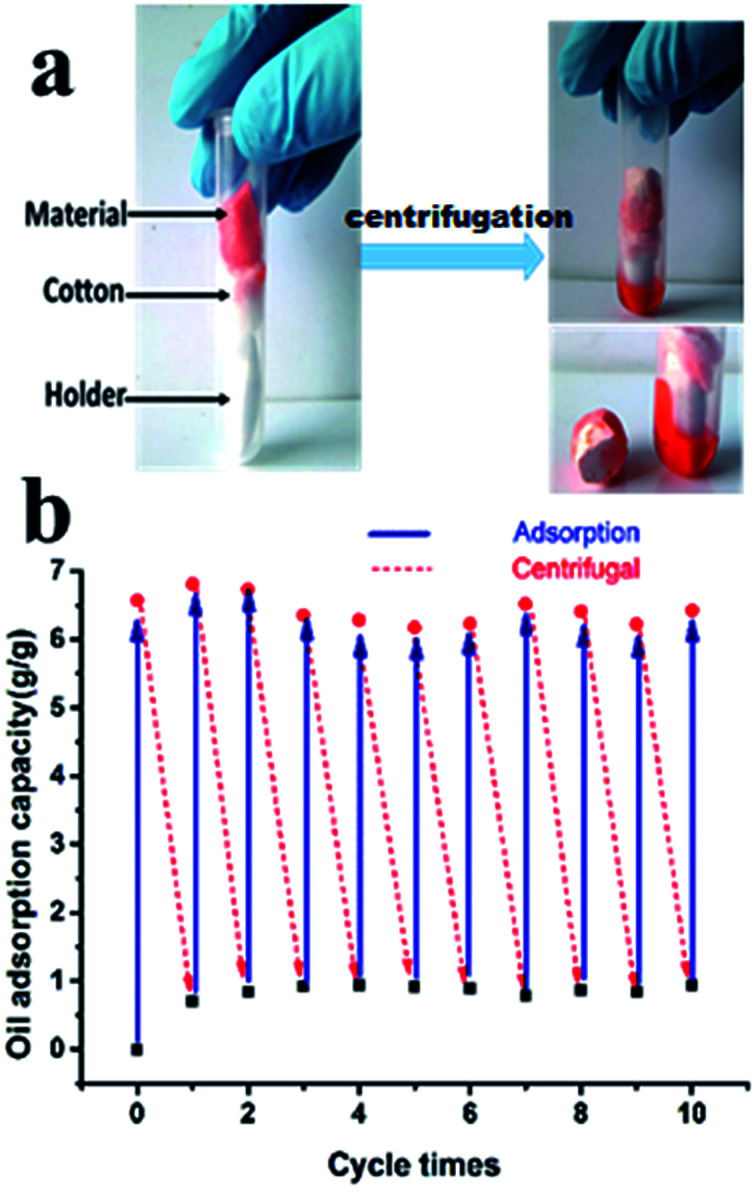
(a) Adsorbed toluene recovery from fluoro-polyHIPE, and (b) recyclability performance of the porous fluoropolymer monolith.

In the recycling experiments, as shown in Fig. 14a, a PTFE-coated magnetic stirrer was used as a hard holder to prevent the cotton and porous material from moving down to the bottom of the tube during or after centrifugation. Cotton was used as a soft holder to put on top of the PTFE-coated magnetic stirrer and, as well as the porous material support that made the oil to pass through. In a reusability experiment, toluene was selected as the means to measure adsorption stability of the porous material. The material was put into a mixture of toluene and water. Once adsorption saturation was attained, the monolithic foam was removed from water and weighed quickly. Then, we centrifuged the mixture for 15 min at 10 000 rpm, as these parameters were proved to be most efficient in the process of recycling. Regenerated polyHIPE foams were then used for the next cycle of toluene adsorption/regeneration. 10 times cycled adsorption experiments were performed and the results showed oil removal rates of more than 85% (Fig. 14b).

A common feature of the results obtained from Fig. 14b was that almost the same amount of residue was left in the monolithic material after each centrifugation and there was only a little decline of oil adsorption capacity even after 10 cycles of adsorption/regeneration. We speculated that this small portion of residual oil (about 15%) was tightly trapped in the tiny pores. After centrifugation, the monolithic foam was regenerated and was directly used for the cycled oil intake experiments. In this environmentally friendly way, oil pollution due to spillage accidents would be eliminated and the precious oil resources would be re-collected at the same time.

## 4. Conclusions

In summary, we systematically studied fluorinated acrylate as raw material by polymerizing a w/o high internal phase emulsion to prepare a series of porous materials. We chose a suitable conventional surfactant, Hypermer B246, for stabilization of water-in-oil fluoro-HIPE to obtain novel high-porosity fluoropolymer foams with tunable morphology (open and closed pores) by investigating concentrations of surfactant, amount of external crosslinker, and aqueous phase volume. Then, fluoropolymer foam was used to adsorb oils from the water surface and also from the bottom. The results presented noticeable adsorption with a very fast rate towards organic oils and repelled water due to the inherent hydrophobic nature of fluorine atoms in the polymer. We also explored the influence of pore structure and surface properties on water contact angle and oil adsorption capacity. Furthermore, the adsorption capacity of the fluoropolymer foams could be approximately 10 times with respect to initial weight. Adding fluoromonomer enabled excellent performance in severe environments such as acidic or alkaline solutions. These fluoropolymer foams with hydrophobic–oleophilic properties and specific interconnected structures will have an excellent potential for oil spillage cleanups.

## Conflicts of interest

Author declares no conflict of interest for this research paper.

## Supplementary Material

RA-008-C8RA00501J-s001

RA-008-C8RA00501J-s002

RA-008-C8RA00501J-s003
