# Supplementary material for: Highly porous and chemical resistive P(TFEMA–DVB) monolith with tunable morphology for rapid oil/water separation
Source: RSC Adv. 2018 Feb 22;8(15):8355–64. doi: 10.1039/c8ra00501j (PMC9078523; doi:10.1039/c8ra00501j)
Supplement: RA-008-C8RA00501J-s003 [file RA-008-C8RA00501J-s003.pdf]

Electronic Supplementary Material (ESI) for RSC Advances.
This journal is © The Royal Society of Chemistry 2018

Supplementary Information

Highly Porous and Chemical Resistive P(TFEMA-DVB) Monolith with Tunable Morphology for Rapid Oil/Water Separation

Xiaozheng Wan[a] †, Umair Azhar [a] †, Yongkang Wang [a], Jian Chen [a], Anhou Xu [a], Shuxiang Zhang[a] *, Bing Geng[a] *

a. *Shandong Provincial Key Laboratory of Fluorine Chemistry and Chemical Materials, School of Chemistry and Chemical Engineering, University of Jinan, Jinan 250022, China. Email: chm_zhangsx@ujn.edu.cn, chm_gengb@ujn.edu.cn*

† *Xiaozheng Wan and Umair Azhar contributed equally to this work*

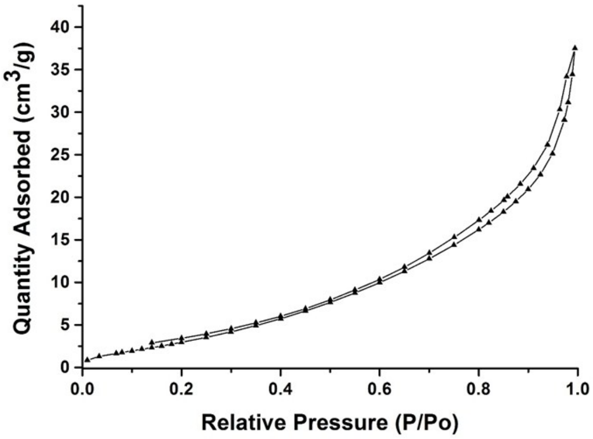

Fig. S1 BET nitrogen adsorption–desorption isotherms of Sample A5.

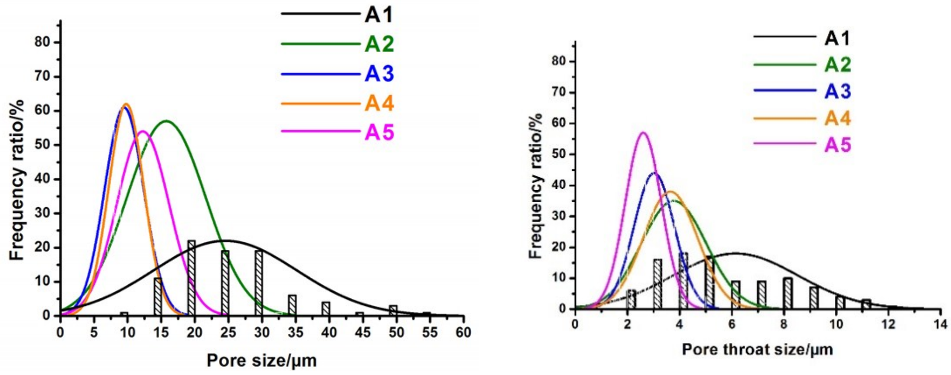

Fig. S2 The normal distribution curve of samples A1-A5 void size and pore throat size analyzed scanning electron microscopy (SEM) images, the samples A5 pore throat size histogram distribution.

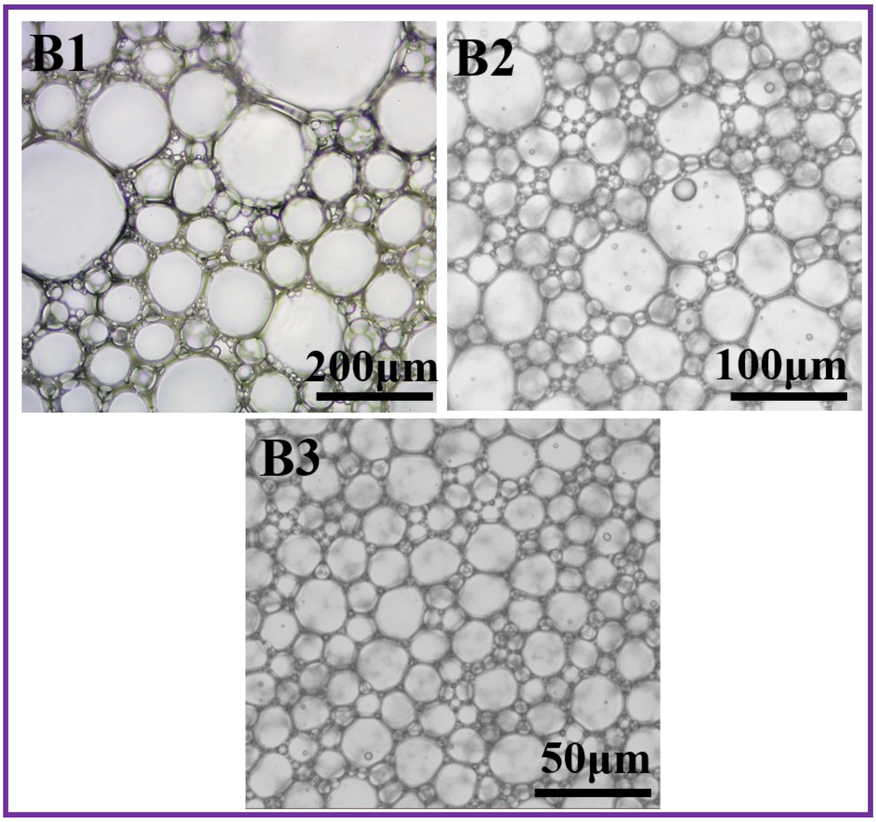

Fig. S3 Optical microscopy images of w/o emulsion with different content of surfactant to oil phase 5 wt% (B1), 10 wt% (B2), 20 wt% (B3).

Table S1 Foam Average Void Diameter (Dv), Throat Diameter (Dt), number of pore throats per bigger pore (Nt) and Density (Dm), Porosity (P), Openness (Op) of macroporous polymers

	Dv [μm]	Dt [μm]	Nt	Dm [g/cm^3]	P [%]	Op [%]	BET Surface Area(m^2/g)
B1	266.4	4.46	—	0.16±0.015	84.32±1.2	—	10.58
B2	127.03	2.75	—	0.1583±0.023	85.73±1.5	—	38.53
B3	14.55	2.31	18±3	0.1492±0.012	86.21±1.3	11.75	19.86

Video S1. Adsorption of toluene (with Oil red O) in water surface by fluorided porous material.

Video S2. Adsorption of dichloromethane (with Oil red O) in water bottom by fluorided porous material.
